# Impact of missing data imputation methods on gene expression clustering and classification

**DOI:** 10.1186/s12859-015-0494-3

**Published:** 2015-02-26

**Authors:** Marcilio CP de Souto, Pablo A Jaskowiak, Ivan G Costa

**Affiliations:** 1 Univ. Orleans, INSA Centre Val de Loire, LIFO EA 4022, Orleans, France; 2 Institute of Mathematics and Computer Sciences, University of São Paulo, São Carlos - SP, Brazil; 3 Center of Informatics, Federal University of Pernambuco, Recife - PE, Brazil; 4 IZKF Computational Biology Research Group, Institute for Biomedical Engineering, RWTH Aachen University Medical School, Aachen, Germany

**Keywords:** Missing data, Imputation, Clustering, Classification, Gene expression

## Abstract

**Background:**

Several missing value imputation methods for gene expression data have been proposed in the literature. In the past few years, researchers have been putting a great deal of effort into presenting systematic evaluations of the different imputation algorithms. Initially, most algorithms were assessed with an emphasis on the accuracy of the imputation, using metrics such as the root mean squared error. However, it has become clear that the success of the estimation of the expression value should be evaluated in more practical terms as well. One can consider, for example, the ability of the method to preserve the significant genes in the dataset, or its discriminative/predictive power for classification/clustering purposes.

**Results and conclusions:**

We performed a broad analysis of the impact of five well-known missing value imputation methods on three clustering and four classification methods, in the context of 12 cancer gene expression datasets. We employed a statistical framework, for the first time in this field, to assess whether different imputation methods improve the performance of the clustering/classification methods. Our results suggest that the imputation methods evaluated have a minor impact on the classification and downstream clustering analyses. Simple methods such as replacing the missing values by mean or the median values performed as well as more complex strategies. The datasets analyzed in this study are available at http://costalab.org/Imputation/.

**Electronic supplementary material:**

The online version of this article (doi:10.1186/s12859-015-0494-3) contains supplementary material, which is available to authorized users.

## Background

The expression level of a gene can be regarded as an estimate of the amount of protein it produces in a given cellular state. Different technologies can be used to measure the expression level of a gene. One of the most important is microarray technology, which allows the simultaneous measurement of the expression levels of thousands of genes [1].

As with many types of experimental data, gene expression data obtained from microarray experiments often contain missing values (MVs) [2-5]. This can occur for several reasons: insufficient resolution, image corruption, fabrication errors, poor hybridization, or contaminants due to dust or scratches on the chip. However, many standard methods for gene expression data analysis, including some classification and clustering techniques, require a complete data matrix as input. Thus, in such a context, methods for handling missing data are needed.

The simplest way of dealing with MVs is to discard the observations that contain them. However, this method is practical only when (1) the data contain a relatively small number of observations containing MVs, or when (2) the analysis of the complete examples will not lead to a serious bias during the inference [6,7]. Neither is viable in the context of microarray data. For example, as pointed out in [5,8], it is common for gene expression data to have up to 5% MVs, which could affect up to 90% of the genes.

Thus, in the microarray setting, instead of repeating the biological experiment (too expensive) or discarding all observations with missing values (negative impact on downstream analyses), many MV imputation methods have been proposed in the literature [4,5]. MV imputation constitutes an entire class of procedures that aim to supply the MVs with estimated ones [7].

The simplest imputation algorithms consist of replacing the MVs by zero or by the corresponding row/column average [9]. However, as discussed in [2], because such methods do not take into account the correlation structure of the data, they tend to perform poorly in terms of estimation accuracy, i.e., how close the estimated value is to the missing value. More complex algorithms that employ gene correlations have been proposed to minimize this problem. These methods include, for example, the weighted *k*-nearest neighbor (WKNN) [2], local least squares (LLS) [10], expectation maximization approach (EM_array) [11] and Bayesian principal component analysis (BPCA) [12] procedures.

Indeed, in the past few years, several papers have presented systematic evaluations of different imputation algorithms [3-5,13-16]. In general, the validation is accomplished by calculating various performance indices about the relation between the imputed and the (known) original values [16]. Initially, most imputation algorithms were evaluated with an emphasis on the accuracy of their imputation (fidelity to the true expression values), using metrics such as the root mean squared error (RMSE).

However, it can be argued that the success of the expression value estimation should be evaluated also in more practical terms [15,16]. For instance, one can consider the ability of the method to preserve the significant genes in the dataset, or its discriminative/predictive power for classification/clustering purposes. In fact, in practical terms of the downstream objective of the experiment (e.g., clustering or classification), if the differences between the outcomes are biologically insignificant, then it is irrelevant whether an MVs imputation improvement is statistically significant or not. Furthermore, in most studies, missing values in microarray data sets are assumed to be missing at random. By following this assumption, the great majority of the aforementioned papers apply imputation methods considering a uniform distribution of missing values on genes and microarrays. This, however, is not a realistic assumption, since missing values tend to arise in a systematic manner in practice [16]. Therefore, these studies are not able to model gene- or array-specific artifacts that induce missing values. Because our analysis is based on an actual MV distribution, we are not susceptible to biases introduced by artificial imputation experiments.

Regarding related work, [17] evaluated the impact of six imputation methods on clustering analyses using eight yeast cDNA microarray datasets, including both time series and steady-state experiments. They performed a cluster analysis on the imputed data using the *k*-means algorithm. The partitions generated after imputation were compared with those obtained originally on the complete datasets. The authors showed that even when there are clear differences in the accuracy of imputation, as assessed using RMSE, such differences could become insignificant when the methods are evaluated in terms of how well they reproduce the original gene clusters, or in their biological interpretations.

With respect to classification, the most closely related research is that in [13], who applied three imputation algorithms, WKNN, LLS and BPCA, to five different cancer gene expression datasets. The classification accuracy was estimated using three different types of classifiers: support vector machine (SVM), *k*-nearest neighbor (*k*NN), and classification and regression tree (CART). They showed that, except for replacement by zeros, the imputation algorithm made little difference in classification.

A limitation of the work in [17] and [13] is that both were based on a small selection of datasets, and presented no statistical evidence for their conclusions. In contrast, we have performed the first systematic comparison of classical missing value imputation methods by following a typical microarray data analysis workflow. More specifically, we analyzed the impact of five missing data imputation methods on several clustering and classification algorithms applied to 12 cancer gene expression datasets. We followed the preprocessing protocol suggested in [18] to do so; that is, we first discarded the genes with missing data for more than some particular number of observations. Next, we replaced the remaining MVs using each of the five data imputation approaches, followed by filtering the genes with low variation. Moreover, for the first time in this field, we used a statistical framework to evaluate whether the differing imputation methods significantly affected the performances of the various classification/clustering methods.

## Results

### Missing value imputation

In the context of gene expression data, MV imputation methods usually fall into two categories [4]. In the first category (“local” methods), the expression information of a missing entry is taken from neighboring genes, where their closeness is determined by a proximity measure (e.g., correlation, or the Euclidean distance). For the second category (“global” methods), dimension reduction techniques are applied to decompose the data matrix and iteratively reconstruct the missing entries. In this paper we use two well-known representatives of the former category: WKNN [2] and LLS [10]. For the latter, we employed the well-established BPCA method [12]. We also provide an additional evaluation of the expectation maximization approach (EM_Array) [11] (see Additional file 1).

We analyzed the effects of these imputation methods, as well as the Mean and Median methods, where MVs are replaced with a simple mean or median, respectively, from known values. Given these methods we conducted two classical downstream analyses for cancer gene expression data: classification and clustering. We used the following experimental design to do so. First, we removed all genes with more than 10 *%* missing values (MV filtering). Next, we imputed the missing values using each of the five methods. Finally, we applied a non-supervised filter to remove genes with little variation between samples. See Section Methods for more details.

Tables 1 and 2 illustrate, respectively, the statistics for the original and the resulting datasets after imputation/non-supervised filtering. An important, initial observation is that none of the datasets have more than 8% missing values (the average is 5.04%, see Table 1). Furthermore, this value drops to an average of 2.32% after the MV filtering. This can be seen as indicative of the upper bound values for experimental settings with artificially imputed missing values. Another interesting observation, as can be seen in Table 2, is that the number of genes remaining after the non-supervised filtering step is quite similar with all the imputation methods. This indicates a minimal impact of the imputation method on the non-supervised filtering step.

**Table 1:** **Cancer datasets with missing values**

					**Original data**			**MV Filtering**		
**Dataset**	**Tissue**	**No. classes**	**Size of classes**	**No. samples**	**No. genes**	**% MV**	**% Genes with MV**	**No. genes**	**% MV**	**% Genes with MV**
alizadeh-2000-v1	Blood	2	21, 21	42	4022	3.25	49.30	3678	2.15	44.56
alizadeh-2000-v2	Blood	3	42, 9, 11	62	4022	4.59	66.93	3369	2.75	60.52
alizadeh-2000-v3	Blood	4	21, 21, 9, 11	62	4022	4.59	66.93	3369	2.75	60.52
bredel-2005	Brain	3	31, 14, 5	179	41472	7.57	43.06	19200	3.25	30.56
chen-2002	Liver	2	104, 75	66	24192	6.04	88.46	22336	2.18	85.46
garber-2001	Lung	4	17, 40,4, 5	110	24192	3.87	67.81	36663	2.23	65.14
lapointe-2004-v1	Prostate	3	11, 39, 19	69	42640	4.56	73.57	35265	2.10	69.26
lapointe-2004-v2	Prostate	4	11, 39, 19, 41	110	42640	4.93	67.16	36663	2.23	60.29
liang-2005	Brain	3	28, 6, 3	37	42640	4.56	73.57	22923	0.82	23.16
risinger-2003	Endometrium	4	13, 3, 19, 7	42	24192	7.97	74.33	8366	0.76	20.76
tomlins-2006	Prostate	5	27, 20, 32, 13, 12	104	8872	4.46	89.34	9936	3.27	80.94
tomlins-2006-v2	Prostate	4	27, 20, 32, 13	92	20001	4.04	84.23	10048	3.34	79.72
**Mean**					**23575**	**5.04**	**70.39**	**17651**	**2.32**	**56.74**

**Table 2:** **Statistics after non-supervised filtering**

	**No. genes (filtering + imputation)**					**No. MV (filtering + imputation)**				
**Datasets**	**BPCA**	**KNN**	**LSS**	**Mean**	**Median**	**BPCA**	**KNN**	**LLS**	**Mean**	**Median**
alizadeh-2000-v1	960	945	962	932	932	1.96	1.91	1.97	1.83	1.83
alizadeh-2000-v2	1075	1050	1081	1030	1030	2.71	2.63	2.72	2.59	2.59
alizadeh-2000-v3	1075	1050	1081	1030	1030	2.71	2.63	2.72	2.59	2.59
bredel-2005	3819	3833	3825	3850	3852	0.81	0.82	0.81	0.84	0.84
chen-2002	2240	2246	2238	2329	2340	2.25	2.24	2.23	2.31	2.32
garber-2001	2563	2540	2578	2584	2603	1.94	1.92	1.95	1.95	1.95
lapointe-2004-v1	4161	4159	4170	4196	4292	1.94	1.92	1.95	1.95	1.95
lapointe-2004-v2	3846	3811	3833	3838	3930	2.50	2.50	2.50	2.53	2.58
liang-2005	2531	2528	2529	2519	2521	2.32	2.29	2.31	2.33	2.37
risinger-2003	942	2074	2078	2073	2073	0.84	0.83	0.84	0.81	0.81
tomlins-2006	2027	2020	2039	2018	2018	2.41	2.40	2.43	2.40	2.40
tomlins-2006-v2	2118	2118	2124	2103	2103	2.37	2.34	2.37	2.34	2.34
**Mean**	**2294**	**945**	**2378**	**2375**	**2018**	**2.06**	**2.04**	**2.07**	**2.04**	**2.05**

### Influence of imputation on clustering/classification

We employed several classification and clustering techniques with the cancer tissues from each imputed dataset. Refer to the Methods section for further details on the methodology. We used SVM, kNN, naive Bayesian classifiers (NB), and decision trees (DT) for the classification experiments. The classification error rate, after leave-one-out cross-validation (LOOCV), was used as our evaluation metric (see Table 3). Importantly, the error rates obtained are all below those of simply predicting the majority class for each dataset.

**Table 3:** **Classification error for different imputation methods (columns) and classificaiton methods (rows)**

	**Datasets**	**Mean**	**Median**	**KNN**	**BPCA**	**LLS**
SVM	alizadeh-2000-v1	9.52	9.52	9.52	9.52	11.90
	alizadeh-2000-v2	0.00	0.00	0.00	0.00	0.00
	alizadeh-2000-v3	6.45	6.45	6.45	6.45	6.45
	bredel-2005	16.00	16.00	16.00	16.00	16.00
	chen-2002	2.23	2.23	2.23	2.23	1.68
	garber-2001	16.67	16.67	18.18	18.18	19.70
	lapointe-2004-v1	1.82	1.82	1.82	1.82	1.82
	lapointe-2004-v2	17.39	18.84	15.94	17.39	20.29
	liang-2005	0.00	0.00	0.00	0.00	0.00
	risinger-2003	21.43	19.05	19.05	19.05	19.05
	tomlins-2006	6.73	7.69	6.73	5.77	5.77
	tomlins-2006-v2	6.52	6.52	6.52	6.52	6.52
KNN	alizadeh-2000-v1	33.33	33.33	33.33	30.95	30.95
	alizadeh-2000-v2	0.00	0.00	0.00	0.00	0.00
	alizadeh-2000-v3	19.35	19.35	17.74	17.74	17.74
	bredel-2005	20.00	20.00	20.00	20.00	20.00
	chen-2002	11.73	11.17	12.29	12.29	12.29
	garber-2001	16.67	16.67	16.67	16.67	18.18
	lapointe-2004-v1	13.64	14.55	14.55	14.55	14.55
	lapointe-2004-v2	33.33	36.23	33.33	33.33	34.78
	liang-2005	2.70	2.70	2.70	2.70	2.70
	risinger-2003	23.81	23.81	23.81	19.05	23.81
	tomlins-2006	20.19	20.19	20.19	20.19	20.19
	tomlins-2006-v2	21.74	21.74	21.74	21.74	21.74
NB	alizadeh-2000-v1	7.14	7.14	7.14	7.14	7.14
	alizadeh-2000-v2	1.61	1.61	1.61	1.61	1.61
	alizadeh-2000-v3	8.06	8.06	6.45	6.45	6.45
	bredel-2005	14.00	14.00	14.00	14.00	14.00
	chen-2002	13.41	12.85	13.41	12.85	13.41
	garber-2001	22.73	24.24	22.73	22.73	22.73
	lapointe-2004-v1	23.64	23.64	23.64	21.82	22.73
	lapointe-2004-v2	31.88	31.88	33.33	33.33	33.33
	liang-2005	18.92	18.92	16.22	16.22	18.92
	risinger-2003	23.81	23.81	23.81	26.19	23.81
	tomlins-2006	15.38	15.38	14.42	14.42	14.42
	tomlins-2006-v2	17.39	17.39	17.39	17.39	17.39
DT	alizadeh-2000-v1	28.57	30.95	11.90	23.81	23.81
	alizadeh-2000-v2	8.06	8.06	14.52	14.52	14.52
	alizadeh-2000-v3	25.81	27.42	25.81	20.97	20.97
	bredel-2005	40.00	38.00	44.00	44.00	44.00
	chen-2002	6.15	7.82	7.26	6.70	6.70
	garber-2001	28.18	21.21	21.21	22.73	19.70
	lapointe-2004-v1	23.64	26.36	23.64	22.73	23.64
	lapointe-2004-v2	28.99	30.43	36.23	36.23	33.33
	liang-2005	8.11	8.11	8.11	8.11	8.11
	risinger-2003	45.24	45.24	54.76	50.00	54.76
	tomlins-2006	40.38	40.38	34.62	36.54	34.62
	tomlins-2006-v2	40.22	40.22	38.04	35.87	34.78

We used k-medoids, hierarchical clustering with average linkage (HC-AL), and hierarchical clustering with complete linkage (HC-CL) for the clustering experiments. Partition quality was evaluated with the corrected Rand (cR) index (see Table 4). All of the partitions generated have cR values larger than 0 for k-medoids; that is, one can claim that they are not random partitions with respect to the ground partition. This also holds true for most of the partitions generated by HC-AL.

**Table 4:** **Corrected Rand index for different imputation methods (columns) and clustering methods (rows)**

	**Datasets**	**Mean**	**Median**	**KNN**	**BPCA**	**LLS**
*k*-medoids	alizadeh-2000-v1	0.5	0.5	0.5	0.5	0.5
	alizadeh-2000-v2	0.89	0.89	0.84	0.89	0.84
	alizadeh-2000-v3	0.65	0.65	0.65	0.65	0.65
	bredel-2005	0.41	0.41	0.41	0.41	0.41
	chen-2002	0.3	0.3	0.3	0.3	0.3
	garber-2001	0.55	0.51	0.54	0.49	0.54
	lapointe-2004-v1	0.42	0.47	0.47	0.44	0.47
	lapointe-2004-v2	0.17	0.17	0.15	0.17	0.15
	liang-2005	0.5	0.5	0.5	0.5	0.5
	risinger-2003	0.45	0.45	0.45	0.47	0.45
	tomlins-2006	0.39	0.39	0.4	0.39	0.4
	tomlins-2006	0.51	0.51	0.51	0.51	0.51
HC-CL	alizadeh-2000-v1	0.04	0.04	0.13	0.13	0.13
	alizadeh-2000-v2	0.54	0.54	0.52	0.40	0.52
	alizadeh-2000-v3	0.38	0.38	0.39	0.47	0.39
	bredel-2005	-0.03	0.07	0.03	0.07	0.03
	chen-2002	-0.01	-0.01	-0.01	-0.01	-0.01
	garber-2001	0.55	0.55	0.55	0.55	0.55
	lapointe-2004-v1	-0.01	-0.01	-0.01	-0.01	-0.01
	lapointe-2004-v2	0.04	0.04	0.04	0.04	0.04
	liang-2005	0.12	0.12	0.12	0.12	0.12
	risinger-2003	0.09	0.09	0.10	0.09	0.10
	tomlins-2006	0.46	0.46	0.39	0.43	0.39
	tomlins-2006	0.39	0.39	0.39	0.39	0.39
HC-AL	alizadeh-2000-v1	0.00	0.00	0.00	0.00	0.00
	alizadeh-2000-v2	0.79	0.79	0.79	0.79	0.79
	alizadeh-2000-v3	0.40	0.40	0.44	0.44	0.44
	bredel-2005	-0.07	-0.08	-0.07	-0.05	-0.07
	chen-2002	-0.01	-0.01	-0.01	-0.01	-0.01
	garber-2001	0.00	0.00	0.00	0.02	0.00
	lapointe-2004-v1	-0.01	-0.01	-0.01	-0.01	-0.01
	lapointe-2004-v2	0.04	0.04	0.04	0.04	0.04
	liang-2005	0.12	0.12	0.12	0.12	0.12
	risinger-2003	0.14	0.14	0.12	0.14	0.12
	tomlins-2006	0.44	0.44	0.41	0.41	0.41
	tomlins-2006	0.56	0.56	0.56	0.56	0.56

As can be clearly seen in Tables 3 and 4, at least within the context of the datasets investigated, the different imputation methods did not have any impact on the performance of the classifiers and the partitions generated. Nonetheless, we applied a statistical test in both cases, to further justify our conclusions.

More specifically, we used the Friedman-Nemenyi test to assess whether there was any statistically significant difference in using a given imputation method for a fixed classification/clustering method [19] — see Table 5 for a summary of the *p*-values. The null hypothesis of equal ranks was not rejected in any of the cases. That is, we have no statistical evidence that any of the imputation methods used have a significant impact on the quality of the classifiers/clustering partitions produced. These conclusions also hold when the EM_Array imputation method is considered in our analysis (see Additional file 1).

**Table 5:** **Summary of the Friedman** ***p*** **-values for the classification and clustering methods**

**Methods**	**Friedman** ***p*** **-value**
DT	0.81
KNN	0.88
NB	0.82
SVM	0.99
HCA-CL	0.95
HCA-AL	0.89
*k*-medoids	0.99

In order to evaluate if the missing values filter (MV filtering) had an impact in our results, we first tried to run the imputation methods without MV filtering. In this context, the implementation of the different imputation methods failed to deal with attributes with 50% or more of missing data. Then, to successfully run most of the methods we had to remove the attributes with more than 40% of missing values. After that, we applied the non-supervised filter. Next, we performed experiments with SVMs (the classification method that in general presented the best results) with the resulting datasets. We also run k-medoids and the hierarchical methods. As for the case of the BPCA, the implementation that we have available failed to run for four datasets. In this context, we performed the hypothesis test without considering it. In all cases, the null hypothesis of equal ranks was not rejected (see Additional file 1).

## Discussion and conclusions

We performed a comprehensive analysis of the impact of five well-known MV imputation methods on three clustering and four classification methods with 12 cancer gene expression datasets. Moreover, we used, for the first time in this field, a statistical framework to evaluate whether distinct imputation methods improve the performance of the clustering/classification methods. Our experimental results indicate that the imputation methods evaluated have a negligible impact on the classification as well as on downstream clustering analysis. Indeed, methods as simple as the Mean and Median, performed as well as more complex strategies such as WKNN and BPCA. As both Mean and Median methods are simple to implement, and have low computational requirements, we recommend that they should be preferred over other methods for classification and clustering tasks with gene expression microarray data.

To help put our results into perspective, the most similar work to ours is that reported in [13,17], as mentioned in the Background section. In [17], the authors compared different missing value imputation methods by following a typical microarray data analysis workflow for clustering. They used the following imputation methods: (1) zero imputation (Zero), in which the MVs are always replaced by a zero; (2) Mean; (3) WKNN; (4) LLS; (5) iterated local least squares (iLLS); (6) support vector regression (SVR); and (7) BPCA. They used eight yeast cDNA microarray datasets for their experiments, including both time series and steady-state trials. They performed a cluster analysis of the imputed data using the *k*-means algorithm. The partitions generated after imputation were compared with those obtained originally on the complete datasets using the average distance between partitions (ADBP).

We employed steady-state cancer gene expression data, unlike [17], who used time series gene expression data. Also, in contrast to [17], who performed clustering with only a partitioning algorithm (*k*-means), we used two hierarchical clustering methods, as well as *k*-medoids. In [17], the authors claimed that more advanced imputation methods, such as BPCA, should be preferable to the Zero or Mean imputation methods. However, they presented no statistical evidence to support these claims. Indeed, visual inspection of the error bars presented in their work indicates a clear disadvantage only for the Zero imputation method.

The work most closely related to ours regarding classification is that in [13], who investigated the effects of the WKNN, LLS and BPCA on the classification performance of SVM, *k*NN, and CART. According to their results, imputation algorithms, except for substitution by zero, did not have any demonstrable impact on classification. The work in [13] was limited to five two-class datasets. We used 12 datasets, with the number of classes varying from two to five. Moreover, in contrast to [13], we also investigated the influence of simple imputation methods such the Mean and the Median in our work.

The fact that gene expression is highly correlated helps to explain why the imputation of expression values close to the original missing value is not required for an analysis based on thousands of genes, such as tissue clustering or classification [20]. This is why our finding that simple methods, such as the Mean and Median, perform comparably to more complex methods makes sense. That is, out of a group of co-expressed (correlated) genes, some genes without missing data could always exist.

We would like to point out that, from a practical point of view, it is common to remove attributes (features) with more than a certain percentage of missing value. As other work in the literature, we set the threshold to 10%. Even in a context of a level of missing values of up to 40%, the statistical test does not point to any significant difference between the imputation methods.

Finally, and of large consequence, of the 32 datasets in the benchmarking cancer gene expression data study in [21], only 12 of them have MVs: a maximum of 9% of MVs per dataset and an average of 5%. Our average number of missing values goes down to 2% after non-supervised filtering (see Tables 1 and 2). This raises questions regarding the conclusions from most gene expression imputation research, which are based on an artificial imputation of at least 20% of the missing values [2,4,5,10,12,13,17]. Our opinion is that in the context of classification and clustering, any conclusions based on more than 5% missing values are mostly irrelevant to gene expression analysis. Moreover, the great majority of the previously mentioned papers apply the imputation methods assuming a uniform distribution of missing values on genes and microarrays. By doing so, they are not able to model gene- or array-specific artifacts inducing missing values. Because our analysis is based on an actual MV distribution, we are not susceptible to biases introduced by artificial imputation experiments.

## Methods

### Datasets and pre-processing

We used gene expression datasets from the benchmark study in [21]. More specifically, 12 cDNA datasets that have MVs were selected. In terms of pre-processing, we followed the protocol suggested in [18,21]. First, we removed all genes that had more than 10 *%* missing entries for the datasets (MV filtering). Next, we applied the different imputation methods (see details below). Finally, we performed a non-supervised filtering to remove genes with low variation between samples. The datasets analyzed in this study are available at http://costalab.org/Imputation/. See Table 1 for (1) dataset names; (2) tissue types; (3) number of classes (cancer types); (4) size of classes; (5) number of samples; and (6) number of genes, in the original data; (7) percentage of missing values (%MV), in the original data; (8) percentage of genes with at least one MV, in the original data; and (9) number of genes, in the dataset after MV filtering; (10) percentage of missing values (%MV), in the dataset after MV filtering; (11) percentage of genes with at least one MV, in the dataset after MV filtering. Table 2 illustrates the number of genes and the percentage of MVs after the application of non-supervised filtering corresponding to the five different imputation methods.

### Imputation methods

This section presents the five imputation methods we evaluated. In what follows, let *n* be the size of the dataset (the number of samples) and *p* be the number of attributes (variables) of each instance.

- WKNN was proposed in [2]. For a given gene with a missing entry, the method is based on finding the *k* closest neighbors. The MV is replaced by the weighted average of the *k*-neighbors, where the weights are proportional to the similarity between samples. WKNN uses Euclidean distances as its similarity measure. In our experiments, following the guidelines in [2], we set the number of neighbors *k* to 20. This method has a computational complexity of $\mathcal {O}(npk)$.
- LLS is based on finding the *k*-nearest genes and estimating the missing values by performing a linear regression on the variable with MVs [10]. In this paper, as in [10], we used the (inverse of) the Pearson correlation coefficient as the similarity measure, and *k*=300. Such a method has a computational complexity of $\mathcal {O}(npk)$.
- BPCA combines a principal component regression with a Bayesian expectation maximization method to estimate the missing values [12]. The method has one important parameter, the number of principal components, to consider. In this paper, as in other papers, we set it to five. The method has a computational complexity of $\mathcal {O}((p +n)c+2 nc)$ and a memory complexity of $\mathcal {O}(np^{2})$, where *c* is the number of components.

Besides these three “advanced” imputation methods, we also applied two very simple ones: Mean and Median, which are based on assigning the mean (or median) value of the gene to the respective missing entries [2]. This is the standard approach in machine learning [7]. They also have a very straightforward implementation, and a computational complexity of $\mathcal {O}(np)$. In the context of gene expression data, these simple methods have often been reported to produce very poor results [2,4,17]. All methods were implemented in R or obtained from the Bioconductor packages impute and pcaMethods. In addition to these five methods we also considered the EM_Array using the implementation provided in [11]. The results concerning this particular method are presented in the Additional file 1.

### Classification and clustering methods

We explored the impacts of several commonly used data imputation methods on the performance of different classification and clustering algorithms by using 12 actual cDNA datasets in this study. More precisely, in terms of classification, we performed an empirical comparison by using four classical supervised learning algorithms [22]: (1) DT, a rule-based system; (2) *k*NN; (3) NB; and (4) SVM.

All of these learning methods were obtained from the WEKA machine learning package [23]. The classifiers were generated and evaluated using a traditional LOOCV [22] procedure. We used the default parameter values for creating the classifiers (e.g., a linear kernel for the SVMs, *k*NN with *k*=1 and Euclidean distance, and a DT with pruning) in the current experiments, based on our previous research [24].

We performed our cluster analysis evaluation employing three clustering algorithms widely applied in the literature of gene expression data: hierarchical clustering with both complete and average linkage [25], and *k*-medoids [26]. We used the inverse of the Pearson correlation coefficient as the similarity measure in all cases. The number of clusters was set to the number of known classes in the corresponding datasets. The ability to recover the actual structure of the data was used as the performance criterion. This was accomplished using the well-known corrected Rand (cR) index [27]. To do so, the cR index compares the actual classes (true partition) of the tissue samples (e.g., cancer types/subtypes) with their cluster assignments.

Finally, all results were statistically compared with the Friedman statistical test for multiple comparisons and the Nemenyi post-test, at the 95% confidence level, according to the procedures described in [19], for both the classification and clustering evaluations. In the case of classification tasks, we used the rate of classification error produced by the LOOCV. For the cluster analysis, we employed the result of the cR.

## Additional file

Additional file 1
**A table formatted for Excel with supplementary results.** The table contains (Table S1) the statistics of all datasets after each filtering and imputation step; (Table S2 and Table S3) the classification error rates for the different classifiers generated for the datasets after 10% (and 40%) of filtering of genes with missing values and missing value imputation; (Table S4 and Table S5) the corrected Rand Index for all clustering methods applied for the datasets after 10% (and 40%) of filtering of genes with missing values and missing value imputation; and (Table S6) *p*-value of the Friedmann-Nemenyi test for experiments, including also the results of the imputation method EM_Array.
